# Supplementary material for: Prospective Multi-Institutional Observational Study of Retreatment with Anti-PD-1/PD-L1 Antibodies in Patients with Non-Small Cell Lung Cancer Previously Treated with Anti-PD-1/PD-L1 Plus Chemotherapy: NJLCG (North Japan Lung Cancer Group) Trial 1901
Source: Cancers (Basel). 2025 May 2;17(9):1551. doi: 10.3390/cancers17091551 (PMC12071100; doi:10.3390/cancers17091551)
Supplement: Supplementary file 1 [file cancers-17-01551-s001.zip › figure legen for Figure S1.pdf]

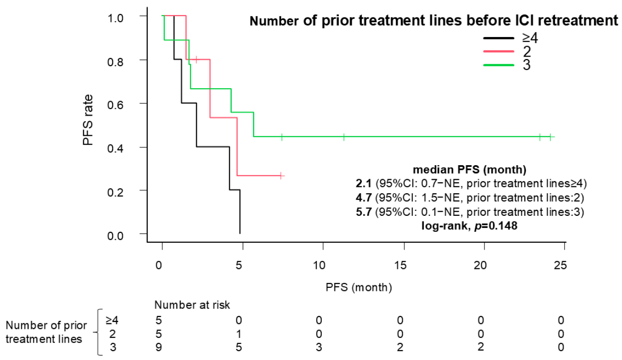


**Figure S1.** PFS according to the number of prior treatment lines before ICI retreatment in patients with an ICI-free interval longer than 11.9 months. Abbreviations: PFS, progression-free survival; ICI, immune checkpoint inhibitor; CI, confidence interval; NE, not estimable.
