# Supplementary material for: Prospective Multi-Institutional Observational Study of Retreatment with Anti-PD-1/PD-L1 Antibodies in Patients with Non-Small Cell Lung Cancer Previously Treated with Anti-PD-1/PD-L1 Plus Chemotherapy: NJLCG (North Japan Lung Cancer Group) Trial 1901
Source: Cancers (Basel). 2025 May 2;17(9):1551. doi: 10.3390/cancers17091551 (PMC12071100; doi:10.3390/cancers17091551)
Supplement: Supplementary file 1 [file cancers-17-01551-s001.zip › Table S1.pdf]

Table S1. Univariate and multivariate analysis of prognostic factors for PFS on ICI retreatment (excluding patients with recurrence after chemoradiotherapy and durvalumab)

	Univariate analysis		Multivariate analysis	
	HR (95%CI)	p-value	HR (95%CI)	p-value
Histology: Non-Sq (vs Sq)	0.40 (0.18–0.85)	0.018	0.65 (0.28–1.52)	0.326
ICI-free interval: >11.9 months (vs ≤11.9 months)	0.39 (0.18–0.84)	0.016	0.33 (0.14–0.77)	0.009
Prior ICI: anti-PD-L1 (vs PD-1)	0.40 (0.14–1.17)	0.094	0.34 (0.10–1.12)	0.075
Retreatment ICI: anti-PD-L1 (vs PD-1)	1.69 (0.76–3.75)	0.19		
Liver metastasis: Positive	2.59 (0.88–7.62)	0.08		
Brain metastasis: Positive	0.92 (0.42–2.03)	0.84		
TPS ≥50% (vs <50%)	0.82 (0.34–1.97)	0.70		
Age, years ≥75 (vs <75)	0.91 (0.34–2.43)	0.85		
Prior ICI discontinuation due to non-PD (vs PD)	1.05 (0.45–2.46)	0.92		
Serum Alb (g/L) ≥3.5	1.07 (0.51–2.24)	0.87		
Serum NLR ≥ 5	0.85 (0.40–1.80)	0.68		

Abbreviations: PFS, progression-free survival; ICI, immune checkpoint inhibitor; PD-1: programmed death 1; anti-PD-L1, anti-programmed death ligand 1; Sq, squamous cell carcinoma; TPS, tumor proportion score; Alb, albumin; NLR, neutrophil-to-lymphocyte ratio; HR, hazard ratio; CI, confidence interval.
